# Delayed Ischemic Hepatocellular Injury Following Cemented Total Hip Arthroplasty: A Case Report Within the Spectrum of Bone Cement Implantation Syndrome

**DOI:** 10.3390/life16030394

**Published:** 2026-02-28

**Authors:** Bogdan Ștefan Boloș, Ruxandra-Cristina Marin, Răzvan Ene, Simona Bianca Vlad, Oana Andreia Coman

**Affiliations:** 1 Department of Orthopaedics, Carol Davila University of Medicine and Pharmacy, 050474 Bucharest, Romania; bogdan-stefan.bolos@drd.umfcd.ro; 2 Department of Ortopedics and Traumatology, Bucharest Emergency Clinical Hospital, 8 Calea Floreasca, 014461 Bucharest, Romania; 3 Department of Pharmacology, Clinical Pharmacology and Pharmacotherapy, Faculty of Medicine, “Carol Davila” University of Medicine and Pharmacy, 050474 Bucharest, Romania; oana.coman@umfcd.ro; 4 Department of ENT, Carol Davila University of Medicine and Pharmacy, 050474 Bucharest, Romania; simona-bianca.vlad@drd.umfcd.ro

**Keywords:** bone cement implantation syndrome, total hip arthroplasty, hepatocellular dysfunction, acute liver injury, methyl methacrylate, multiorgan failure, case report

## Abstract

Bone cement implantation syndrome (BCIS) is classically associated with acute intraoperative cardiopulmonary disturbances during cemented arthroplasty. However, accumulating clinical observations suggest that its systemic manifestations may extend beyond the immediate peri-cementation period. Hepatic involvement remains rarely reported and is poorly characterized, particularly in frail elderly patients with limited physiological reserve. We report the case of an 82-year-old woman who developed severe but reversible ischemic acute liver failure with concomitant acute kidney injury following cemented total hip arthroplasty. A brief peri-cementation episode of hypotension and mild hypoxemia was followed, within the early postoperative period, by abrupt elevation of aminotransferases (AST 4980 IU/L; ALT 3120 IU/L), coagulopathy (INR ≥ 1.5), transient neurological alteration compatible with early hepatic encephalopathy, severe acute kidney injury, and new-onset atrial fibrillation. An extensive diagnostic evaluation excluded viral, autoimmune, toxic, biliary, vascular, infectious, and structural causes of liver injury. The clinical and biochemical profile was consistent with ischemic hepatocellular injury occurring in the context of systemic hypoperfusion. Management consisted of supportive intensive care focused on hemodynamic stabilization, respiratory support, rhythm control, metabolic management, and close laboratory monitoring, resulting in complete hepatic, renal, and neurological recovery. This case describes a rare presentation of ischemic acute liver failure with multiorgan involvement following cemented total hip arthroplasty. The temporal association with transient peri-cementation hypotension and hypoxemia suggests a possible delayed systemic manifestation within the spectrum of BCIS, even in the absence of overt intraoperative collapse. Although causality cannot be established, the clinical course underscores the importance of careful postoperative evaluation in vulnerable patients who experience perioperative hemodynamic disturbances.

## 1. Introduction

Total hip arthroplasty (THA) occupies a central role in contemporary orthopedic practice, not only because of its high clinical success rates but also due to its ever-increasing global demand. Long-term registry data consistently report implant survival exceeding 90–95% at 10–15 years following primary THA, with sustained improvements in pain relief, mobility, and quality of life across diverse patient populations, establishing THA as one of the most effective surgical interventions in modern medicine. Current estimates suggest that THA procedures will surpass 2.8 million annually by 2030. A U.S. Medicare-based projection reported an annual growth rate of approximately 5.2% for primary THA, with procedure volumes expected to rise substantially through 2040 and beyond [1,2]. This expanding demand is driven primarily by population ageing and the increasing burden of hip osteoarthritis and fragility-fracture surgery [3].

In many high-income countries, THA ranks among the most frequently performed inpatient surgical procedures, reflecting both improved access to care and continued advances in implant design, anesthetic management, and perioperative pathways [2].

Within this context, the choice of prosthesis fixation remains particularly relevant in elderly patients with osteoporotic bones. Cemented fixation continues to be preferred in this population, as large registry-based and observational studies demonstrate superior immediate mechanical stability and significantly lower rates of early periprosthetic femoral fracture compared with uncemented stems. Reported reductions in periprosthetic fracture risk in older adults range from approximately 30% to over 50% with cemented fixation, particularly in patients with poor bone quality or fragility fractures [4,5]. These advantages support the continued widespread use of cemented THA in high-risk elderly populations despite the recognized risk of cement-related systemic complications.

Despite the widespread use of cemented THA in older individuals, emerging evidence suggests that systemic complications related to bone cement may be more complex and heterogeneous than previously recognized. Historically, attention has focused primarily on cardiopulmonary compromise, such as intraoperative hypotension and embolic phenomena during cementation. However, contemporary anesthesia data and advanced monitoring techniques increasingly indicate that cement-related physiological disturbances may involve multiple organ systems, particularly in frail patients with limited physiological reserve [6,7].

In hip-fracture populations, cemented arthroplasty is frequently selected to facilitate immediate fixation and early mobilization in osteoporotic bone. However, these patients often have limited cardiopulmonary and microvascular reserves. As a result, even brief peri-cementation hemodynamic disturbances may lead to clinically meaningful downstream organ injury, even when intraoperative instability appears mild and rapidly corrected. Furthermore, BCIS may manifest beyond the moment of cementation, with postoperative features such as transient hypoxia or confusion reported in older patients undergoing cemented arthroplasty. This broader clinical spectrum highlights the need for careful postoperative surveillance in high-risk patients [8,9].

One organ system that has received comparatively little attention in this context is the liver. The liver plays a central role in xenobiotic metabolism and systemic inflammatory regulation, and perioperative hepatic vulnerability is increasingly recognized in major orthopedic surgery, particularly in association with hemodynamic instability and inflammatory activation. Recent clinical observations indicate that even transient episodes of perioperative hypoperfusion may precipitate substantial hepatocellular injury in older adults with reduced physiological reserve [10].

In the specific setting of THA, hepatic dysfunction may arise through several mechanisms. Ischemia related to systemic hypoperfusion represents the most immediate and biologically plausible pathway in the perioperative setting. However, additional contributors have been described, including exposure to bone-cement components, immune activation, and implant-derived substances. Experimental data suggest that methyl methacrylate may induce hepatocellular mitochondrial dysfunction and microvascular inflammatory changes, although direct clinical evidence in humans remains limited. Implant-derived metal ion dissemination, particularly cobalt and chromium, has also been reported, mainly in association with metal-on-metal bearings or mechanically assisted crevice corrosion. However, these mechanisms are uncommon, typically develop over longer timeframes, and are less consistent with acute postoperative presentations. Consequently, transient systemic hypoperfusion is currently considered the most plausible mechanism underlying acute perioperative organ Injury, with bone cement implantation syndrome (BCIS) providing a unifying pathophysiological framework [6,11,12].

Nevertheless, the development of acute, severe hepatocellular injury following THA remains rare and requires careful documentation when it occurs. The biochemical presentation may mimic other etiologies, including ischemic hepatitis, drug-induced liver injury, viral hepatitis, or sepsis. Recent surgical cohorts show that postoperative liver dysfunction, although uncommon, may present with heterogeneous biochemical patterns that can complicate early diagnosis [13].

Early recognition of postoperative hepatic dysfunction is essential, as even moderate biochemical abnormalities may reflect clinically significant hepatocellular injury in elderly patients with reduced hepatic reserve. Clinically relevant postoperative liver injury is commonly defined by aminotransferase elevations exceeding three times the upper limit of normal or by impaired hepatic synthetic function, including an international normalized ratio (INR) ≥1.5 or rising bilirubin levels [14,15]. Recent perioperative cohort studies report postoperative liver enzyme abnormalities in approximately 5–15% of patients undergoing major non-hepatic surgery, with higher rates observed in elderly and high-risk populations [13].

Importantly, surgery-related hepatic dysfunction is associated with increased short-term morbidity and mortality, with 30-day mortality risks reported to be two- to fourfold higher in patients with elevated liver enzymes. Large contemporary surgical population analyses further show that perioperative organ injury, including hepatic dysfunction, is associated with increased complications, prolonged hospitalization, and reduced survival, particularly when multiorgan dysfunction is present [16,17].

Diagnostic differentiation between ischemic, toxic, and inflammatory hepatic injury can be challenging in the perioperative setting, as these conditions often share overlapping clinical and laboratory features. Current perioperative recommendations therefore emphasize a structured diagnostic approach, including continuous hemodynamic assessment, infection screening, targeted imaging when indicated, evaluation of coagulation and bilirubin parameters, and careful review of medications and surgery-related exposures [18].

Given the diverse but individually rare mechanisms of hepatic injury associated with cemented THA, as well as the frequently overlooked link between bone cement and hepatic dysfunction, delineating the clinical spectrum of this complication is essential. By presenting a detailed chronological account of postoperative deterioration, diagnostic workup, and recovery, the aim of this case report is to describe an unusual presentation of acute, reversible ischemic hepatocellular injury with multiorgan dysfunction following cemented total hip arthroplasty, and to discuss its potential relationship with delayed systemic manifestations of bone cement implantation syndrome in a frail elderly patient.

## 2. Case Description

### 2.1. Patient Presentation and Preoperative Assessment

**Figure 1:**
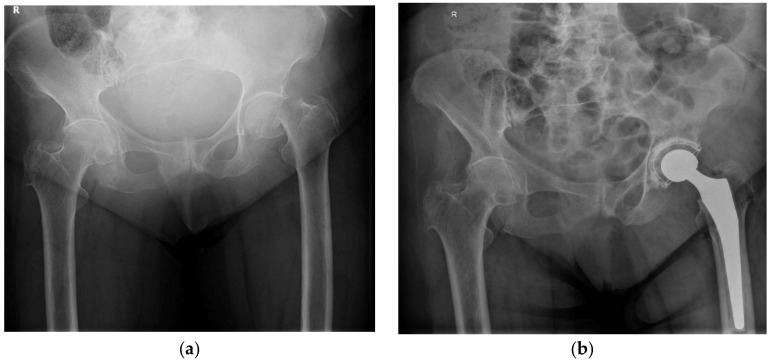
Radiographic evaluation of the left hip. (**a**) admission anteroposterior radiograph showing a displaced intracapsular femoral neck fracture (Garden IV); (**b**) postoperative radiograph after cemented total hip arthroplasty.

An 82-year-old woman was admitted with an intracapsular femoral neck fracture classified as Garden IV [19] characterized by complete displacement of the fracture fragments and loss of cortical continuity between the femoral head and femoral neck, following a low-energy fall from standing height (Figure 1a).

On admission, the patient appeared frail but oriented, with stable vital signs and no abnormalities on physical examination aside from painful limitation of the left hip. Her functional status reflected complete immobility secondary to the displaced femoral neck fracture sustained after a same-level accidental fall. Preoperative anesthetic risk stratification classified the patient as American Society of Anesthesiologists (ASA) physical status III [20].

Cardiopulmonary evaluation revealed a blood pressure of 112/72 mmHg, rhythmic and symmetric heart sounds without murmurs, and preserved peripheral pulses. Chest radiography demonstrated mild aortic unfolding of the cardiac silhouette, blunting of the right costophrenic angle, and band-like atelectatic opacities in the right lower lung field. Electrocardiography demonstrated sinus rhythm at 75 beats per minute with isolated ventricular extrasystoles and no ST–T segment abnormalities.

The patient’s medical history was notable only for well-controlled hypertension. She denied alcohol consumption and had no known history of liver disease. Preoperative laboratory investigations obtained during outpatient evaluation revealed normal hepatocellular and cholestatic enzymes (AST 22 IU/L, ALT 18 IU/L, GGT 30 IU/L), a normal coagulation profile (INR 1.0), and a white blood cell count of 6.8 × 10^3^/µL. Previous viral hepatitis screening had reportedly been negative, although complete documentation was unavailable.

### 2.2. Surgical Procedure

The patient underwent cemented THA under combined spinal–epidural anesthesia with sedation. A standard posterior surgical approach to the hip was employed. The implanted prosthetic components included a cobalt–chromium femoral stem and head, a polyethylene acetabular cup, and a polyethylene femoral canal plug. Bone fixation was achieved using polymethylmethacrylate cement (Optipac Refobacin 40 and 80), mixed under vacuum to optimize polymerization and reduce cement porosity (Figure 1b). Perioperative antibiotic prophylaxis was administered with cefuroxime 1.5 g intravenously 

The intraoperative course remained largely uneventful until cementation and insertion of the femoral component, when the patient experienced a transient hypotensive episode, with systolic blood pressure decreasing from 130 mmHg to approximately 75 mmHg over two minutes, accompanied by mild hypoxia (oxygen saturation nadir of 92% on FiO_2_ 0.40). Hemodynamic stabilization was promptly achieved with a 5 mg intravenous bolus of ephedrine and increased oxygen supplementation. No cardiovascular collapse, cardiac arrest, or need for cardiopulmonary resuscitation occurred, and no severe bone cement implantation syndrome features were evident intraoperatively.

Intraoperatively, the patient received a total of 1500 mL of Ringer’s solution, with an estimated blood loss of 300 mL. No significant acidosis or hypothermia occurred. Urine output remained low but present, consistent with age-related renal reserve.

### 2.3. Early Postoperative Course and Clinical Deterioration

Upon admission to the intensive care unit (ICU) on postoperative day (POD) 0, the patient was initially supported with vasopressors and mechanical ventilation. No sedatives, benzodiazepines, or opioids were administered after ICU arrival, allowing reliable neurological assessment. Although early postoperative parameters appeared transiently stabilized, urine output was markedly reduced, raising early concern for evolving end-organ hypoperfusion.

Within the first postoperative hours, the clinical course followed a biphasic pattern, with progressive hypotension requiring escalating circulatory support and worsening oliguria. By POD 1, laboratory evaluation revealed acute and severe hepatocellular injury accompanied by impaired bilirubin handling and evolving coagulopathy, indicating early hepatic involvement in the context of systemic physiological stress. Inflammatory markers increased in parallel, while preserved fibrinogen levels argued against disseminated intravascular coagulation and supported predominant hepatic synthetic dysfunction. Acute liver failure (ALF) was defined as an international normalized ratio (INR) ≥1.5 in the presence of any degree of hepatic encephalopathy in a patient without preexisting chronic liver disease.

On POD 2, the patient exhibited further systemic deterioration with the onset of new high-frequency atrial fibrillation, coinciding with persistent hemodynamic instability. Despite this progression, neurological examination demonstrated preserved responsiveness, with mild somnolence but intact command-following. Renal function declined rapidly to near-anuric acute kidney injury, while aminotransferases, international normalized ratio (INR), and inflammatory markers continued to rise, reflecting escalating multiorgan dysfunction consistent with impaired systemic oxygen delivery rather than isolated organ-specific pathology [21].

Arterial blood gas analysis showed no major metabolic derangements beyond a mild respiratory alkalosis attributable to ventilatory management, and non-contrast cranial computed tomography excluded acute intracranial pathology. At this stage, the differential diagnosis included ischemic hepatitis, drug- or toxin-induced liver injury, acute viral hepatitis, biliary obstruction, autoimmune hepatitis, and sepsis-related multiorgan dysfunction. However, the temporal sequence, initial postoperative stabilization followed by delayed systemic deterioration, strongly suggested a transient perioperative insult with downstream effects, temporally linked to cementation and consistent with a delayed systemic manifestation within the spectrum of bone cement implantation syndrome (BCIS). Severity scoring (APACHE II 17, SOFA 8) confirmed significant acute physiological derangement and evolving multiorgan failure [22,23].

### 2.4. Postoperative Trajectory of Hepatic and Systemic Abnormalities and Corresponding Diagnostic Investigations

**Figure 2:**
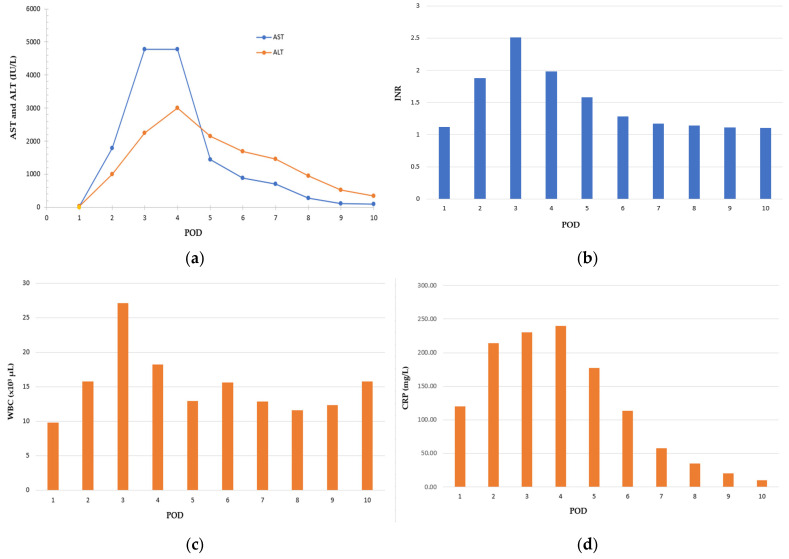
Postoperative laboratory trends by postoperative day. (**a)** aspartate aminotransferase and alanine aminotransferase; (**b**) international normalized ratio; (**c**) white blood cell count; (**d**) C-reactive protein. AST, aspartate aminotransferase; ALT, alanine aminotransferase; INR, international normalized ratio; WBC, white blood cell count; CRP, C-reactive protein; POD, postoperative day.

The temporal evolution of leukocyte count, inflammatory markers, coagulation parameters, and hepatocellular injury is illustrated in Figure 2. Serum aminotransferases (AST and ALT, IU/L) increased abruptly, peaking on POD 4, followed by a progressive decline over the first postoperative week (Figure 2a). The international normalized ratio (INR) reached a maximum on POD 3 and gradually normalized as hepatic synthetic function recovered (Figure 2b). White blood cell count (WBC, ×10^3^/µL) showed early postoperative leukocytosis with steady resolution and no secondary rise, in the absence of microbiological evidence of infection (Figure 2c). C-reactive protein (CRP, mg/L) exhibited a monophasic postoperative elevation peaking on POD 4 with a sustained decline thereafter (Figure 2d). Taken together, these trajectories supported a noninfectious course compatible with transient perioperative hypoperfusion-related hepatic injury rather than sepsis or immune-mediated pathology.

Given the abrupt onset and severity of liver dysfunction, an extensive etiologic evaluation was undertaken. Abdominal ultrasound demonstrated a liver of normal size with smooth contour and homogeneous echotexture, without focal lesions. The portal vein and common bile duct were of normal caliber with preserved flow. The gallbladder and pancreas were not visualized. The spleen measured 9.8 cm and appeared homogeneous. Both kidneys were normal, without calculi or pyelocaliceal dilation. No intraperitoneal free fluid was present, and mild aerocolia was noted. Doppler ultrasound, contrast-enhanced abdominal CT, transthoracic echocardiography, CT pulmonary angiography, and extensive microbiological testing excluded vascular, obstructive, cardiac, thromboembolic, infectious, and structural causes of hepatic dysfunction. An extensive diagnostic evaluation was performed to exclude alternative etiologies of acute hepatic dysfunction, as summarized in Table 1.

Taken together, the evolution of hepatic and systemic injury, along with the comprehensive diagnostic evaluation, supports the interpretation of a transient perioperative hypoperfusion event resulting in reversible ischemic liver injury rather than an infectious or immune-mediated process. The evaluation further excluded obstructive, vascular, infiltrative, and structural causes of hepatic dysfunction, and the overall diagnostic profile consistently argued against viral hepatitis, autoimmune disease, drug toxicity, biliary obstruction, cardiac dysfunction, thromboembolic disease, and sepsis. This systematic exclusion of alternative etiologies strongly reinforces a perioperative ischemic mechanism as the most plausible cause of the acute hepatic injury.

### 2.5. Intensive Care Unit Management

Based on the diagnostic profile, management focused on supportive care tailored to the expected course of ischemic liver injury. The early postoperative course was characterized by hemodynamic instability requiring vasopressor support, severe acute kidney injury with initial anuria followed by polyuric recovery, and marked hepatocellular injury with coagulopathy. Clinical deterioration peaked during POD 1–3. From POD 4 onward, progressive hemodynamic stabilization, improving renal function, and normalization of hepatic parameters were observed, accompanied by resolution of arrhythmia and discontinuation of vasopressor therapy. These changes marked the transition to the recovery phase and allowed transfer from the ICU on POD 7. The temporal evolution of hemodynamic status, organ function, and major supportive interventions is summarized in Table 2.

Supportive management included hemodynamic stabilization with fluid resuscitation and vasopressor therapy, rhythm control for atrial fibrillation with intravenous amiodarone followed by oral maintenance therapy, systemic anticoagulation, glycemic control with insulin therapy, and respiratory support with supplemental oxygen. Stress hyperglycemia peaked at 601 mg/dL before gradual normalization.

Neurological status improved progressively from POD 3. Renal function began to recover by POD 5, with increasing urine output and improving biochemical parameters. Throughout the ICU stay, blood and urine cultures remained negative, and inflammatory markers declined steadily. No clinical or microbiological evidence of infection emerged, further supporting a non-septic etiology of hepatic failure.

### 2.6. Final Diagnostic Considerations and Clinical Outcomes

The overall clinical, biochemical, and imaging profile was most consistent with acute liver failure secondary to ischemic hepatocellular injury occurring as a delayed manifestation within the spectrum of BCIS. This diagnosis was supported by abrupt aminotransferase elevation, coagulopathy (INR > 1.5), hepatic encephalopathy, the temporal relationship with cementation, and systematic exclusion of alternative etiologies.

The patient demonstrated steady clinical improvement. Neurological function normalized by POD 4. By POD 5, she was hemodynamically stable without vasopressor support, fully awake, and breathing spontaneously. Liver enzymes declined to <100 IU/L and INR normalized by POD 6, while renal function partially recovered by POD 7. She was transferred to the orthopedic ward on POD 7 and discharged home on POD 14 with complete normalization of liver biochemical parameters.

At three-month follow-up, the patient remained asymptomatic with no evidence of chronic liver dysfunction. Orthopedic recovery was satisfactory, with a well-functioning prosthesis and independent ambulation using a cane.

## 3. Discussion

While bone cement implantation syndrome (BCIS) is classically characterized by acute cardiopulmonary disturbances, the present case illustrates that the liver may also represent a vulnerable end-organ target of perioperative oxygen-delivery failure associated with cemented arthroplasty. In this elderly, previously non-cirrhotic patient, a brief episode of peri-cementation hypotension and mild hypoxemia was followed by ALF with multiorgan dysfunction, consistent with ischemic hepatocellular injury secondary to transient systemic hypoperfusion. Increasing evidence indicates that BCIS is not solely an intraoperative cardiopulmonary event but a systemic syndrome with potential downstream organ consequences, particularly in frail hip-fracture patients with limited physiological reserve [6].

Notably, the intraoperative insult in this case was limited in duration and severity, yet the postoperative course followed a biphasic trajectory characterized by initial stabilization followed by delayed deterioration. This pattern highlights how even transient peri-cementation hemodynamic disturbances may be clinically amplified in vulnerable individuals. Contemporary geriatric hip-fracture data demonstrate that frailty markedly increases postoperative morbidity and mortality, and that short-lived hypotensive episodes may translate into disproportionate organ injury in very old patients [24].

The diagnostic profile strongly supported an ischemic mechanism. Key features included a temporally related hypotensive–hypoxic event, abrupt and marked aminotransferase elevation, acute coagulopathy with neurological alteration, concurrent acute kidney injury, and complete biochemical and clinical recovery following supportive management. Extensive etiological evaluation excluded viral, infectious, biliary, vascular, autoimmune, and toxic causes. These findings align with established clinical patterns of hypoxic or ischemic hepatitis rather than primary inflammatory or toxic liver injury [25,26,27].

Neurologically, the patient exhibited mild postoperative somnolence with preserved responsiveness and intact command-following. In the absence of sedatives and in the presence of hepatic dysfunction and coagulopathy, this presentation is compatible with early-grade hepatic encephalopathy, although contributions from critical illness cannot be entirely excluded. The rapid neurological recovery supports an acute and reversible process rather than primary neurological pathology [28].

Laboratory kinetics further reinforced a hypoxic–ischemic injury pattern. Aminotransferases rose abruptly, peaked around postoperative days (POD) 3–4, and declined rapidly thereafter, paralleling clinical improvement and occurring in the absence of infectious or obstructive findings. This temporal profile is characteristic of ischemic hepatic injury and supports impaired oxygen delivery as the dominant pathogenic mechanism [26,27]. Recent guideline-based reviews emphasize that ALF due to circulatory failure may demonstrate rapid biochemical reversal when oxygen delivery is restored early, provided alternative etiologies are systematically excluded [14].

Beyond hepatic involvement, this case further supports the concept of BCIS as a systemic perioperative syndrome. Observational data have documented multiorgan dysfunction, including renal and hepatic failure, in severe BCIS presentations, underscoring systemic hypoperfusion as a central pathogenic mechanism [29]. In the present patient, hepatic injury and acute kidney injury emerged concurrently and improved in parallel with hemodynamic stabilization, a pattern typical of global oxygen-delivery failure rather than isolated organ-specific pathology. Recent case-based evidence has also described delayed or biphasic BCIS presentations, reinforcing the possibility of postoperative deterioration despite an initially controlled intraoperative course [30].

BCIS is now recognized as a clinical spectrum ranging from mild hypoxia and hypotension to cardiovascular collapse. Although intraoperative features in this case were limited and consistent with a lower-grade phenotype, the subsequent systemic deterioration illustrates that intraoperative grading alone may not fully predict downstream risk in frail patients [31].

Certain patient populations are particularly susceptible to BCIS-related complications due to reduced physiological reserve and limited tolerance to abrupt cardiopulmonary stress. Advanced age, frailty, hip-fracture surgery, osteoporosis, and higher anesthetic risk status (ASA class III–IV) are consistently associated with increased vulnerability to hypoxia, hypotension, arrhythmias, and secondary organ hypoperfusion. Preexisting cardiopulmonary disease, pulmonary hypertension, right ventricular dysfunction, chronic diuretic therapy, and relative hypovolemia further impair compensatory responses to embolic and hemodynamic stress during cementation. Procedure-related factors may further increase risk, particularly those that elevate intramedullary pressure and embolic load, such as forceful cement pressurization, unvented femoral canals, long-stem or revision prostheses, and extensive intramedullary instrumentation. In addition, perioperative management factors (inadequate preoperative volume optimization, insufficient hemodynamic monitoring in high-risk patients, delayed recognition of early hypoxia or hypotension, and failure to anticipate cardiovascular instability during cement insertion) may exacerbate BCIS severity. Recognition of both patient-related vulnerability and modifiable procedural and perioperative factors is essential for risk stratification and early intervention [6,7,8,31,32].

Pathophysiologically, BCIS involves intramedullary embolization of fat, marrow, and cement particles, acute increases in pulmonary vascular resistance, right ventricular dysfunction, and the release of vasoactive mediators, culminating in systemic oxygen-delivery impairment. Impaired tissue perfusion and oxygen delivery are well-recognized triggers of mitochondrial dysfunction and oxidative stress, which amplify inflammatory signaling and contribute to systemic organ injury; comparable mechanisms have been described in other acute critical conditions characterized by hypoxia-mediated cellular damage [33]. The absence of radiologically detectable thromboembolism does not exclude microembolization or mediator-driven pulmonary vascular changes sufficient to impair systemic perfusion in elderly patients with limited physiological reserve [7]. The hepatic injury pattern observed (massive, transient aminotransferase elevation with modest hyperbilirubinemia and rapid resolution) closely matches established definitions of ischemic hepatitis and ischemic ALF. Large cohort studies confirm that ischemic ALF carries one of the highest probabilities of spontaneous survival when hemodynamic instability is corrected early, consistent with the favorable outcome observed in this patient [34,35].

AKI followed a similarly reversible trajectory, transitioning from oligo-anuria to polyuric recovery in parallel with hepatic improvement. This coordinated recovery pattern further supports a shared systemic insult rather than fixed structural organ injury [36].

From a cardiological perspective, the development of new-onset atrial fibrillation during peak hemodynamic instability likely reflects myocardial stress related to BCIS-associated systemic and pulmonary vascular perturbations. Arrhythmias are recognized features of BCIS and typically reflect physiological stress rather than isolated primary rhythm disorders [8,37]. The temporal concurrence of atrial fibrillation, vasopressor dependence, hepatic dysfunction, and renal failure at the point of maximal instability further supports a unified systemic process driven by impaired oxygen delivery [32].

Despite growing recognition of BCIS as a systemic syndrome, detailed descriptions of fulminant yet reversible ALF with severe AKI following cemented arthroplasty remain rare [17,38].

This case therefore supports a low threshold for early postoperative surveillance of hepatic and renal function in frail patients who experience even brief peri-cementation hypotension or hypoxemia. From a perioperative management perspective, integrating hepatic synthetic markers, especially INR, into early postoperative assessment alongside cardiopulmonary monitoring may facilitate earlier recognition of evolving multiorgan dysfunction. Because early biochemical abnormalities may represent sensitive indicators of global hypoperfusion rather than isolated hepatic disease, proactive laboratory surveillance may support timely escalation of care and prevent progression to irreversible injury in high-risk patients.

Finally, the complete recovery observed highlights the impact of prompt multidisciplinary care, including early ICU admission, hemodynamic optimization, arrhythmia management, and structured exclusion of alternative etiologies. Early supportive management remains a key determinant of survival in ischemic ALF, a conclusion reinforced by the present clinical course [15].

### Clinical Implications

From a clinical perspective, this case highlights the importance of extending postoperative surveillance beyond cardiopulmonary parameters in frail elderly patients undergoing cemented hip arthroplasty. It demonstrates that even transient perioperative disturbances in systemic oxygen delivery, when rapidly corrected and not associated with overt intraoperative collapse, may precede clinically relevant downstream organ dysfunction. In this context, early postoperative abnormalities in hepatic function and aminotransferase kinetics may serve as sensitive indicators of global hypoperfusion rather than isolated hepatic disease. Recognition of this reversible ischemic injury pattern may support timely diagnostic prioritization, prevent premature etiologic attribution, and facilitate escalation to supportive, perfusion-oriented management. Emphasizing early pattern recognition and coordinated postoperative surveillance may therefore help reduce the risk of progression toward irreversible multiorgan injury in high-risk surgical populations.

However, these clinical considerations should be interpreted in light of several limitations. As a single observational case, this report cannot establish causality between bone cement implantation and the development of acute ischemic liver failure, despite a compelling temporal association and biological plausibility. Certain diagnostic elements, such as serial lactate or ammonia measurements, invasive hemodynamic monitoring, or histological confirmation, were unavailable owing to the patient’s frailty and the emergent clinical setting, limiting precise quantification of the severity and duration of hypoperfusion. In addition, the individual impact of specific supportive interventions cannot be independently determined and should be interpreted cautiously in the absence of comparative data.

Despite these limitations, the case offers several strengths that enhance its clinical relevance. The detailed temporal documentation of biochemical, hemodynamic, and neurological changes enabled a coherent reconstruction of the progression from perioperative insult to transient multiorgan dysfunction and recovery. The diagnostic evaluation was comprehensive and aligned with contemporary standards for acute liver failure, allowing robust exclusion of alternative etiologies. By integrating current evidence on BCSI and ischemic hepatic injury, this report contributes to the limited literature on delayed, reversible hepatic failure in this setting. The favorable outcome observed reinforces the potential impact of early recognition, multidisciplinary intensive care management, and timely supportive intervention in selected high-risk patients.

## 4. Conclusions

This case describes a rare occurrence of ischemic acute liver failure with concomitant acute kidney injury following cemented total hip arthroplasty in a frail elderly patient. The temporal association with a brief peri-cementation hypotensive–hypoxic episode suggests a possible delayed systemic manifestation within the spectrum of bone cement implantation syndrome, even in the absence of overt intraoperative collapse. While causal inference cannot be established, the clinical course underscores the importance of careful postoperative evaluation in vulnerable patients, particularly when transient intraoperative hemodynamic disturbances occur. Early identification of hepatic and renal dysfunction and supportive management may be associated with clinical recovery in selected cases.

## Figures and Tables

**Table 1:** Diagnostic evaluation for acute hepatic dysfunction.

Diagnostic Category	Test/Imaging	Result
Viral hepatitis	HAV IgM, HBV surface antigen and core IgM, HCV antibody and RNA, HEV IgM	All negative
Other viral infections	CMV IgM, EBV VCA IgM	Negative
Structural hepatobiliary imaging	Abdominal ultrasound	Normal liver size and texture; normal portal vein and common bile duct; gallbladder and pancreas not visualized; spleen 9.8 cm; kidneys normal; no free fluid; mild aerocolia
Vascular/hepatic blood flow	Doppler ultrasound	Patent hepatic and portal veins; no thrombosis; no biliary obstruction
Advanced abdominal imaging	Contrast-enhanced abdominal CT	No hepatic infarcts; normal hepatic arteries and veins; no biliary dilation
Cardiac evaluation	Transthoracic echocardiography	Normal systolic function; no heart failure
Thromboembolic evaluation	CT pulmonary angiogram	No pulmonary embolism
Microbiological testing	Blood and urine cultures	No growth

HAV IgM, hepatitis A virus immunoglobulin M; HBV, hepatitis B virus; HCV, hepatitis C virus; HEV, hepatitis E virus; RNA, ribonucleic acid; CMV, cytomegalovirus; EBV VCA IgM, Epstein–Barr virus viral capsid antigen immunoglobulin M; CT, computed tomography.

**Table 2:** ICU clinical course and supportive interventions by postoperative day.

POD	Hemodynamics	Respiratory Support	Renal Function	Hepatic Function	Major Interventions	Clinical Status
Early deterioration phase (POD 0–3)						
0	Norepinephrine 0.41 µg/kg/min	Mechanical ventilation → O_2_ 6 L/min	Anuria	Rising AST/ALT	Fluid resuscitation	ICU admission
1	Norepinephrine 0.64 µg/kg/min	O_2_ 4–6 L/min	20 mL/24 h	Marked cytolysis, INR ↑	Insulin therapy (glucose peak 601 mg/dL)	Persistent hypotension
2	Norepinephrine 0.3 µg/kg/min	O_2_ 4–6 L/min	1300 mL/ 24 h	Ongoing liver injury	Amiodarone 150 mg, IV vials (2 vials diluted in 50 mL 0.9% sodium chloride), administered as continuous intravenous infusion at 6 mL/hour.	New atrial fibrillation
3	Norepinephrine 0.12 µg/kg/min	O_2_ 4–6 L/min	3900 mL/ 24 h	INR peak	Unfractionated heparin infusion	Initial stabilization
Recovery phase (≥POD 4)						
4	Norepinephrine 0.05 µg/kg/min	O_2_ 4–6 L/min	2650 mL/ 24 h	Improving enzymes	Supportive care continued	Hemodynamic improvement
5	No vasopressors	O_2_ 4–6 L/min	eGFR 42.7 mL/min/ 1.73 m^2^	Improving INR	Enoxaparin 40 mg twice daily	Stable sinus rhythm
6	Stable	O_2_ 2–3 L/min	eGFR 73 mL/min/ 1.73 m^2^	Near normalization	Oral amiodarone 200 mg/day	Clinical recovery
7	Stable	Room air	Normal urine output	AST/ALT <100 IU/L; INR normalized	Transfer from ICU	Clinical stabilization

POD, postoperative day; O_2_, oxygen supplementation; eGFR, estimated glomerular filtration rate; AST, aspartate aminotransferase; ALT, alanine aminotransferase; INR, international normalized ratio.

## Data Availability

The original contributions presented in this study are included in the article. Further inquiries can be directed to the corresponding authors.

## Abbreviations

The following abbreviations are used in this manuscript:

AF	Atrial fibrillation
AKI	Acute kidney injury
ALF	Acute liver failure
ALP	Alkaline phosphatase
ALT	Alanine aminotransferase
ANA	Antinuclear antibodies
aPTT	Activated partial thromboplastin time
ASMA	Anti–smooth muscle antibodies
AST	Aspartate aminotransferase
BCIS	Bone cement implantation syndrome
BP	Blood pressure
CMV	Cytomegalovirus
CRP	C-reactive protein
CT	Computed tomography
EBV	Epstein–Barr virus
eGFR	Estimated glomerular filtration rate
FiO_2_	Fraction of inspired oxygen
GCS	Glasgow Coma Scale
HAV	Hepatitis A virus
HBV	Hepatitis B virus
HCV	Hepatitis C virus
HEV	Hepatitis E virus
HR	Heart rate
ICU	Intensive care unit
INR	International normalized ratio
MAP	Mean arterial pressure
MMA	Methyl methacrylate
O_2_	Oxygen
POD	Postoperative day
SpO_2_	Peripheral oxygen saturation
THA	Total hip arthroplasty
UFH	Unfractionated heparin
ULN	Upper limit of normal
WBC	White blood cell count
